# Boromycin has Rapid-Onset Antibiotic Activity Against Asexual and Sexual Blood Stages of *Plasmodium falciparum*

**DOI:** 10.3389/fcimb.2021.802294

**Published:** 2022-01-14

**Authors:** Laís Pessanha de Carvalho, Sara Groeger-Otero, Andrea Kreidenweiss, Peter G. Kremsner, Benjamin Mordmüller, Jana Held

**Affiliations:** ^1^ Institute of Tropical Medicine, University of Tübingen, Tübingen, Germany; ^2^ Centre de Recherches Médicales de Lambaréné, Lambaréné, Gabon; ^3^ German Center for Infection Research (DZIF), partner site Tübingen, Tübingen, Germany; ^4^ Department of Medical Microbiology, Radboud University Medical Center, Nijmegen, Netherlands

**Keywords:** antibiotics, *Plasmodium falciparum*, *Plasmodium knowlesi*, tetracyclines, delayed death effect, boromycin, macrolides

## Abstract

Boromycin is a boron-containing macrolide antibiotic produced by *Streptomyces antibioticus* with potent activity against certain viruses, Gram-positive bacteria and protozoan parasites. Most antimalarial antibiotics affect plasmodial organelles of prokaryotic origin and have a relatively slow onset of action. They are used for malaria prophylaxis and for the treatment of malaria when combined to a fast-acting drug. Despite the success of artemisinin combination therapies, the current gold standard treatment, new alternatives are constantly needed due to the ability of malaria parasites to become resistant to almost all drugs that are in heavy clinical use. *In vitro* antiplasmodial activity screens of tetracyclines (omadacycline, sarecycline, methacycline, demeclocycline, lymecycline, meclocycline), macrolides (oleandomycin, boromycin, josamycin, troleandomycin), and control drugs (chloroquine, clindamycin, doxycycline, minocycline, eravacycline) revealed boromycin as highly potent against *Plasmodium falciparum* and the zoonotic *Plasmodium knowlesi*. In contrast to tetracyclines, boromycin rapidly killed asexual stages of both *Plasmodium* species already at low concentrations (~ 1 nM) including multidrug resistant *P. falciparum* strains (Dd2, K1, 7G8). In addition, boromycin was active against *P. falciparum* stage V gametocytes at a low nanomolar range (IC_50_: 8.5 ± 3.6 nM). Assessment of the mode of action excluded the apicoplast as the main target. Although there was an ionophoric activity on potassium channels, the effect was too low to explain the drug´s antiplasmodial activity. Boromycin is a promising antimalarial candidate with activity against multiple life cycle stages of the parasite.

## 1 Introduction

Malaria remains one of the world’s most serious public health problems, especially in tropical and subtropical areas, with approximately 241 million cases and 627,000 deaths in 2020 (World Health Organization, 2021). Intracellular parasites of the genus *Plasmodium* cause the disease, and among the six species that can infect humans *P. falciparum, P. vivax, P. malariae, P. ovale wallikeri*, *P. ovale curtisi*, and the zoonotic species *P. knowlesi* (Rutledge et al., 2017; Grignard et al., 2019), *P. falciparum* is the most virulent (World Health Organization, 2021). *P. knowlesi*, described originally as a simian parasite, was recognized in 2000 as a zoonotic species that can infect humans (White, 2008). Although *P. knowlesi* occurs mainly in Malaysia, counting 13,612 malaria cases between 2017 to 2020 (Hu et al., 2021), it can cause severe malaria in humans (Amir et al., 2018) and is phylogenetically closer to other human pathogenic *Plasmodium* species (*P. malariae, P. vivax*, and *P. ovale species*) than *P. falciparum* (Rutledge et al., 2017; van Schalkwyk et al., 2019).

*Plasmodium* parasites have a complex life cycle that includes mosquito and vertebrate hosts (Siciliano and Alano, 2015). Clinical symptoms of malaria appear during the replication of parasites in the erythrocytic cycle (lasting approximately 24 h for *P. knowlesi* and 48 h for *P. falciparum*) in the human host (van Biljon et al., 2018). These symptoms can develop into life-threatening complications such as severe anemia, liver and kidney failure and cerebral malaria if not treated properly (Milner et al., 2014). During each replication cycle, a small number of asexual parasites starts a different genetic program and develops into the transmissible sexual form, named gametocytes (Portugaliza et al., 2020). Gametocytes are non-dividing parasite stages with low metabolic activity whose immature stages are hidden in the bone marrow while the mature stage (stage V) circulates in the blood stream (Gardiner and Trenholme, 2015)

Currently, the mainstay *P. falciparum* malaria treatments are artemisinin-based combination therapies (ACT) (World Health Organization, 2021). Artemisinin and its derivatives are fast-acting and rapidly reduce the asexual parasite load but have a short half-life (2-4 h for artemisinin and <1 h for artesunate) (Benakis et al., 1997). They are combined to a slow-acting partner drug with longer half-life to clear remaining parasites, and to protect artemisinin against resistances. Nonetheless, reports of *P. falciparum* with delayed clearance after artesunate or ACT treatment are accumulating, especially from the Greater Mekong Region where resistances to the partner drugs are also present (Pluijm et al., 2019; WWARN K13 Genotype-Phenotype Study Group, 2019). More recently first reports of kelch13 mutated parasites with a slow clearance have also appeared from Africa (Balikagala et al., 2021). This poses a threat to the existing malaria treatment options (Straimer et al., 2015; Nsanzabana, 2019). In addition, the current ACT drugs are inactive against mature *P. falciparum* gametocytes (stage IV and V). In low transmission areas is recommended to give additionally a single dose of primaquine, except in children younger than six months, pregnant and breastfeeding women with infants younger than six months, to prevent transmission of the parasite, but these recommendations are so far not strictly followed in all areas (World Health Organization, 2021).

The interest in using antibiotics for malaria treatment appeared due to the emergence of resistant parasites to the former mainstay drug chloroquine. Tetracyclines were the first antibiotics used to treat uncomplicated malaria, dating back to the 1950s (Imboden et al., 1950; Grande et al., 1956). Currently, doxycycline and the combination of sulfadoxine-pyrimethamine are used for malaria prevention of travelers and risk groups in malaria-endemic regions, respectively. In addition, clindamycin combined with quinine is used to treat pregnant women during the first trimester or combined with artesunate or quinine to treat uncomplicated malaria when an ACT is unavailable (World Health Organization, 2021).

Tetracyclines and macrolides, both naturally produced by *Streptomyces*, have shown promising antiplasmodial activities as reviewed before (Gaillard et al., 2015; Gaillard et al., 2016; de Carvalho et al., 2021). Most antibiotics with antiplasmodial activity target the apicoplast; a plastid-like organelle derived from endosymbiotic bacteria responsible for the biosynthesis of isoprenoid precursors (Yeh and DeRisi, 2011). Antibiotics can be fast or slow-acting drugs, depending on whether they target the treated parasites or act on their progeny, causing the so-called delayed death effect (Dahl and Rosenthal, 2007). Commonly, tetracyclines belong to the latter group (Gaillard et al., 2015; de Carvalho et al., 2021), and novel tetracyclines have shown superior antiplasmodial activity (e.g. eravacycline and tigecycline IC_50_s at 14 and 38 nM, respectively (Held et al., 2010; Koehne et al., 2021) versus doxycycline and tetracycline IC_50_s at 241 and 340 nM, respectively) (Koehne et al., 2021). Some macrolides such as ivermectin (Mendes et al., 2017; de Carvalho et al., 2019), borrelidin (Otoguro et al., 2003; Ishiyama et al., 2011), and kitasamycin (also called leucomycin) (Ekland et al., 2011) had a fast/first cycle *in vitro* growth inhibitory activity against *P. falciparum* (IC_50_ values of ~ 100 nM, 1.8 nM and 50 nM, respectively). However, the molecular targets of these agents in *Plasmodium* species are not known.

**Figure 1:**
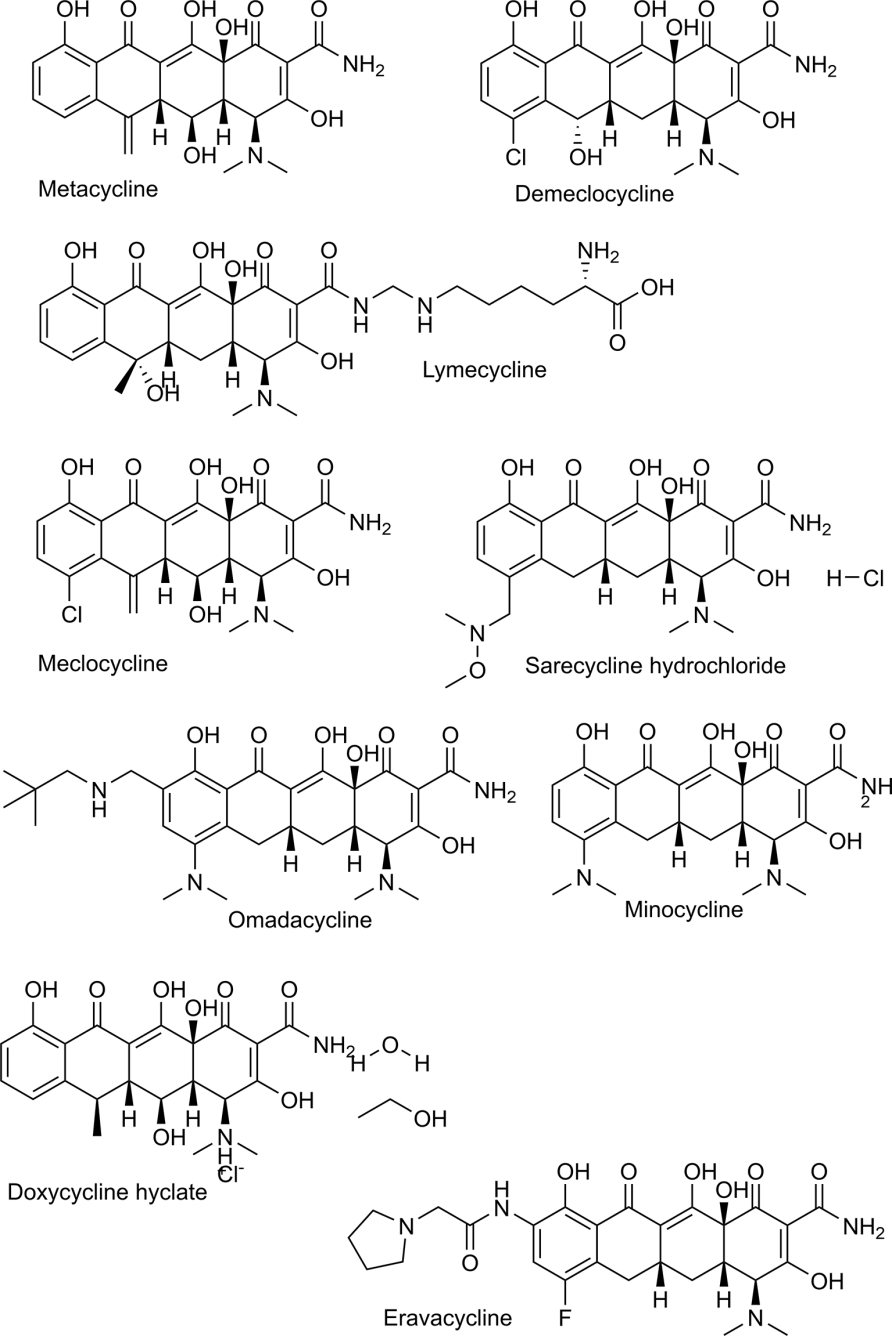
Chemical structures of tetracyclines tested in this study.

**Figure 2:**
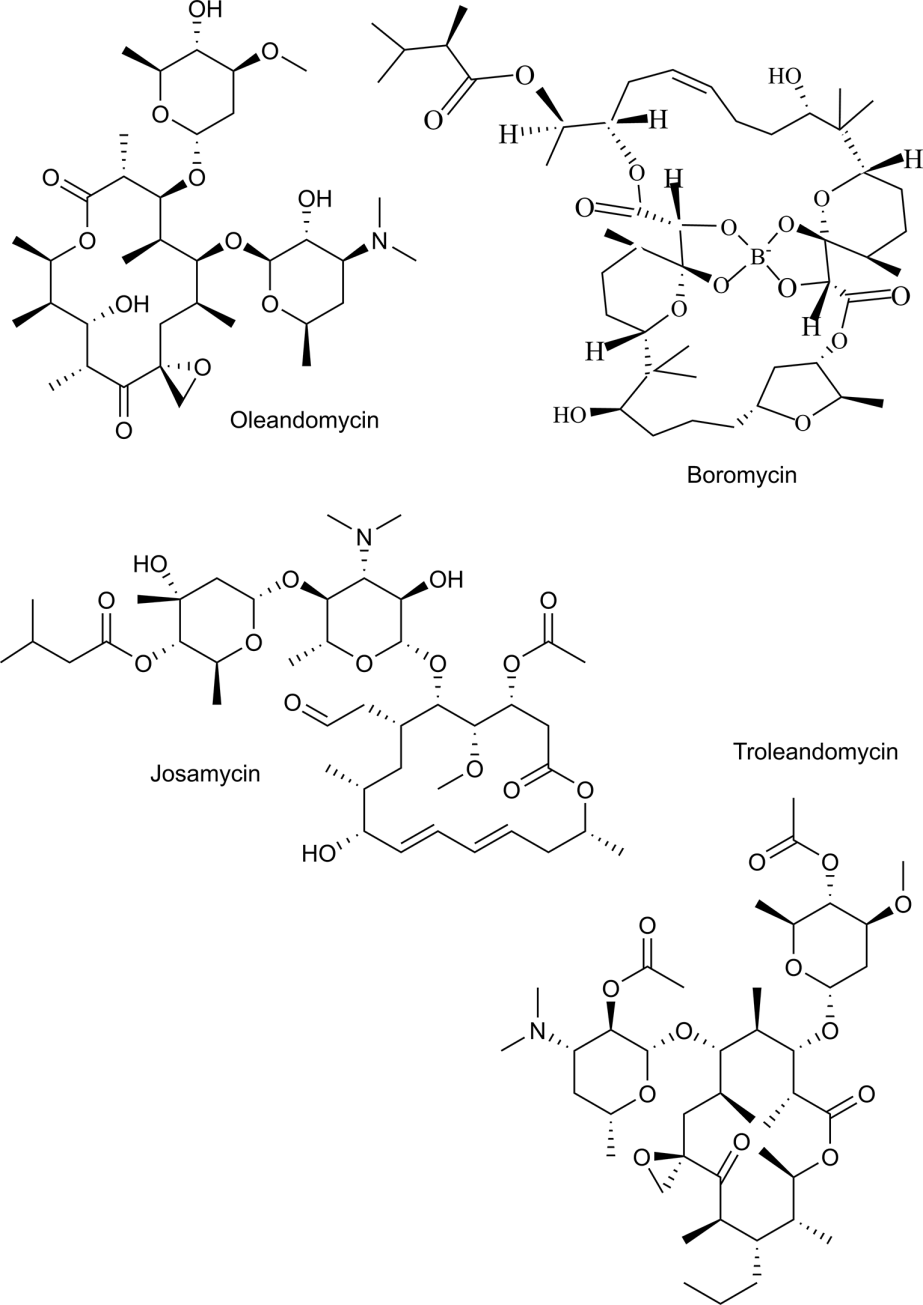
Chemical structures of macrolides tested in this study.

This study aimed to identify antibiotics that are potent and fast acting against *P. falciparum*. Therefore, we evaluated the antiplasmodial effect of two recently marketed tetracyclines, named sarecycline, and omadacycline and some “old” tetracyclines in clinical use named demeclocycline, meclocycline, lymecycline, chlortetracycline and methacycline (**Figure 1**). In addition, the macrolides oleandomycin, troleandomycin, josamycin, and boromycin were evaluated (**Figure 2**). Tetracyclines with known antiplasmodial activity were used as controls. We identified boromycin to be a highly active and fast acting antibiotic. Boromycin is a polyether-macrolide antibiotic produced by *Streptomyces antibioticus* and was the first boron-containing compound found in nature (Dunitz et al., 1971). Previous studies showed a potent effect of boromycin against Gram-positive bacteria, HIV virus and protozoan parasites (Kohno et al., 1996; Moreira et al., 2016; Abenoja et al., 2021). Due to its low nanomolar activity in our screening, we further assessed its antiplasmodial profile also including transmission stages and investigated whether its potential mode of action involves the apicoplast as described for antibiotics in general (Dahl and Rosenthal, 2007) or ionophoric activity on potassium channels as shown before for boromycin (Moreira et al., 2016). In addition, we evaluated its *in vitro* activity against another *Plasmodium* species (*P. knowlesi*).

## 2 Materials and Methods

### 2.1 Chemicals

All tested drugs were first dissolved in their respective solvents and subsequently further diluted in complete culture medium to have the desired final concentration (**Supplementary Table 1** and **Supplementary Information Figures 1, 2**).

### 2.2 Parasite *In Vitro* Culture

#### 2.2.1 Plasmodium Asexual Stages

*P. falciparum* laboratory strains 3D7 (chloroquine-sensitive, provided by BEI resources, MRA-102), Dd2 (multidrug-resistant, provided by BEI resources, MRA-150), K1 (multidrug-resistant, provided by BEI resources, MRA-159), 7G8 (chloroquine resistant, provided by BEI resources, MRA-154) and NF-54 (chloroquine sensitive, provided by Sanaria, a vigorous gametocyte producer), and *P. knowlesi* laboratory strain A1-H.1 (kindly provided by Robert Moon of LSHTM, London, UK under a MTA by the Francis Crick institute) were cultivated *in vitro* as described before (Moon et al., 2013; de Carvalho et al., 2019). Briefly, the parasites were maintained in complete culture medium consisting of RPMI-1640 (Sigma-Aldrich) supplemented with 1M N-2-hydroxyethylpiperazine-N-2-ethane sulfonic acid (HEPES) solution (2.4% v/v) (Sigma-Aldrich), 200 mM L-glutamine (Gibco), 50 µg/ml gentamicin (Gibco), and 10% of AlbuMax II solution containing RPMI, HEPES, NaHCO_3_, D-Glucose, hypoxanthine and 50 g/l of AlbuMax II (0.5% wt/vol in culture medium) at 2.5% hematocrit. The cultures were maintained at 5% CO_2_, 5% O_2_, at 37°C, with a change of medium every two days. *P. falciparum* parasites were synchronized by magnetic column separation prior to the assays (Ribaut et al., 2008). *P. knowlesi* was cultivated as described for *P. falciparum*, with minor modifications. Parasites were cultivated in complete culture medium as detailed above supplemented with 5% human AB serum. Shortly before the growth inhibition assays, *P. knowlesi* were synchronized by density centrifugation with Nycodenz solution (Progen) to recover mature schizonts as described before (van Schalkwyk et al., 2017). The Nycodenz solution was prepared according to the manufacturer’s instruction and kept protected from the light at 4°C and warmed before using.

The asexual growth inhibition assays were performed with 3D7 and A1-H.1, while NF-54 was used for gametocyte assays.

#### 2.2.2 *P. falciparum* Sexual Stages

*P. falciparum* gametocyte culture was performed as described previously (de Carvalho et al., 2019) with minor modifications. The gametocyte culture was initiated with synchronized ring-stage NF54 parasites at 6% hematocrit and 0.3% parasitemia. Complete culture medium (as described above) was supplemented with 5% human serum and changed daily without parasite dilution for 14 days. The medium was doubled when parasitemia reached 5%, and 50-mM *N*-acetyl-d-glucosamine (MP Biomedicals GmbH) was added between day 11 and day 14 to remove asexual stages. On day 15, mature gametocytes were purified with a Nycodenz and magnetic column separation (MACS) to remove erythrocytes and concentrate the gametocyte population.

### 2.3 Growth Inhibition Assays With Asexual Parasites

Growth inhibition assays of asexual stages of *P. falciparum* were performed as described before (Noedl et al., 2005). Briefly, the drugs (**Supplementary Table 1**) were distributed in a 3-fold serial dilution in 96-well plates. The highest concentration of the solvent (<0.1%) did not interfere with parasite growth. Synchronized ring-stage parasites were diluted to a parasitemia of 0.05% with O Rh+ erythrocytes in complete culture medium and seeded at a hematocrit of 1.5% in a total volume of 225 μl per well. Plates were subsequently incubated at 5% CO_2_, 5% O_2_, at 37°C before the plates were frozen and thawed three times. The growth inhibition of *P. falciparum* was measured through an enzyme-linked immunosorbent assay (ELISA) for histidine-rich protein 2 (HRP2) using a microplate reader (CLARIOstar BMG Labtech) (excitation filter: 450 nm).

Growth inhibition assays with *P. knowlesi* were performed as described previously with minor modifications (van Schalkwyk et al., 2019). Briefly, boromycin and chloroquine were distributed in a 3-fold serial dilution in 96-well plates. Synchronized ring-stage parasites were diluted to a parasitemia of 0.5% (a higher parasitemia compared to the HRP2 based assay is needed due to the differences in the limit of detection and therefore optimal assay conditions) with O Rh+ erythrocytes and subsequently seeded at a hematocrit of 2% and complete culture medium without human AB serum but double (20%) AlbuMax II solution in a total volume of 225 μl per well. The growth inhibition of *P. knowlesi* was measured with SYBR Green-I (ThermoFisher) using CLARIOstar (BMG Labtech) (490 nm excitation filter) as HRP2 is not present in this species. For comparison, growth inhibition of boromycin and chloroquine – treated *P. falciparum* was measured using the same methodology.

*P. falciparum* was incubated with the drugs for 3 and 6 days to assess the onset of activity. Both time points were assessed because many antibiotics target only the progeny of the treated parasites, causing a delayed death effect that is measured only after the second replication cycle (after 6 days). Chloroquine and clindamycin were used as positive controls for 3- and 6-day assays, respectively.

To observe the parasite morphology, *P. falciparum* strain 3D7 and Dd2 were incubated with boromycin at 1 nM and for comparison also 3D7 with chloroquine at 8 nM for 6 h for visual observation of drug effect by light microscopy. Thin blood smears were prepared, fixed in 100% methanol for 10 seconds, and stained with Giemsa (Merck) solution (5%) for 20 minutes. The slides were washed and observed under a Leica DMBL microscope, and pictures were taken using a ProgRes C10 camera and software (Jenoptik), at 100X magnification. Three biological replicates were prepared.

### 2.4 *In Vitro* Activity Against Mature Gametocytes of *P. falciparum*

Drug sensitivity assays against mature (stage V) *P. falciparum* gametocytes were performed as described previously (Lelièvre et al., 2012; de Carvalho et al., 2019). Briefly, the drugs were precoated in a 3-fold dilution in 96-well plates. The concentration of the solvent did not interfere with parasite growth. Epoxomicin and methylene blue (**Supplementary Table 1**) were used as positive control. Subsequently, the previously purified mature gametocytes were seeded to the plates (50,000 gametocytes/well) and incubated at 37°C in 5% CO_2_ and 5% O_2_. After 48 h, the ATP production was measured by the BacTiterGlo assay (Promega), according to the manufacturer’s instructions. Finally, the results were quantified using a luminometer (LUmo; Autobio). In addition, to visually inspect the effect of boromycin on gametocytes thin blood smears of untreated and boromycin (8 nM) and epoxomicin (15 nM) - treated gametocytes (50,000 gametocytes/well), were prepared, fixed in 100% methanol for 10 seconds, and stained with Giemsa (Merck) solution (5%) for 20 minutes. The slides were washed and observed under a Leica DMBL microscope, and pictures were taken using a ProgRes C10 camera and software (Jenoptik), at 100X magnification.

### 2.5 Cytotoxicity Assay

HepG2 cells (obtained from ATCC; HB-8065), a human hepatocyte carcinoma cell line, were maintained in DMEM medium (Sigma-Aldrich) supplemented with 10% of inactivated foetal bovine serum (Sigma-Aldrich), 200 mM L-glutamine (Gibco), 12 mL of HEPES buffer (Gibco), and 50 µg/ml penicillin/streptomycin solution (Gibco). Trypsin (Gibco) was used to detach the cells when they reached a semi-confluent layer.

Cytotoxicity was evaluated using the neutral red assay as described previously (Mishra et al., 2021). Briefly, 300,000 cells were seeded in supplemented Dulbecco´s Modified Eagle´s Medium (DMEM) medium as described above to 96-well plates. After 24 hours, the cells were incubated with a twofold serial dilution of the respective drug diluted in supplemented DMEM medium for an additional 24 hours. The highest concentration of the solvent (<1%) did not interfere with cell viability. Subsequently, the drug-containing medium was replaced by a supplemented DMEM medium with 1.5% of neutral red, and the cells were incubated for an additional 3 h at 37°C. Cells were then washed with phosphate buffered saline (pH 7.2), and 100 µL of freshly prepared lysing buffer (50% methanol, 49% distilled water, and 1% acetic acid) were added to the plates. Subsequently, the cells were shaken for 10 min and the absorption was measured at a wavelength of 540 nm using CLARIOstar (BMG Labtech).

### 2.6 Isopentenyl Pyrophosphate Trilithium Salt (IPP) Rescue Assay

Growth inhibition assays were prepared as described in section *Parasite In Vitro Culture* with modifications to assess the specificity of boromycin for the inhibition of the isoprenoid precursor biosynthetic pathway (MEP/DOXP/non-mevalonate pathway) found in the apicoplast (Uddin et al., 2018). Briefly, the parasites (*P. falciparum* strain 3D7) were incubated with boromycin and clindamycin (positive control) and supplemented with IPP (Sigma) at 200 µM for 6 days as described before (Uddin et al., 2018). Since biosynthesis of isoprenoid precursors is the only required function of the apicoplast, supplementation with IPP can rescue treated parasites if the apicoplast is the main target of the drug (Uddin et al., 2018). The assay was done with and without IPP supplementation on the same plate and results were directly compared.

### 2.7 Ionophoric Activity Assay

Previously, boromycin was described to act as a potassium ionophore in *Bacillus subtilis* and *Mycobacterium tuberculosis* (Pache and Zaehner, 1969a; Moreira et al., 2016). So herein, growth inhibition assays complemented with KCl or MgCl_2_ (negative control) were performed to evaluate if this is also the case in *P. falciparum*. To define a subtoxic concentration of MgCl_2_ and KCl for *P. falciparum* a standard growth inhibition assays with serial dilutions of these compounds were performed as described in section *Parasite In Vitro Culture*. The concentration of 220 µg/mL was chosen as no signs of toxicity were seen and first inhibitory effects were observed with 440 µg/mL (**Supplementary Figure 1**) Subsequently, a growth inhibition assay was prepared with a 2-fold dilution of boromycin with the addition of the sub-toxic concentration of either MgCl_2_ (Sigma) or KCl (Sigma) to all wells. The assay was run in parallel to a standard growth inhibition assay of boromycin without supplementation of MgCl_2_ or KCl as control, and the obtained IC_50_s were compared.

### 2.8 Analysis

All assays were performed at least three times in duplicates. Individual IC_50_ values were determined by nonlinear regression analysis of log concentration-response curves, using the drc v3.0-1 package (Ritz and Streibig, 2005) of R v4.1.2 (R Core Team, 2014). Mean IC_50_ values and standard deviations (SDs) were calculated for each growth inhibition assay using Excel. The chemical structures of the drugs were drawn using ChemDraw 19.0. The graphical presentations and statistical analyses (unpaired t-test) were done with GraphPad Prism v8.

## 3 Results

### 3.1 Activity of Antibiotics Against Asexual Stages of *P. falciparum* and *P. knowlesi*

*P. falciparum* was exposed to 15 antibiotics (tetracyclines, macrolides) and chloroquine (control drug) for 3 and for 6 days. This allowed to assess the drug´s inhibitory effect on the first and second parasite replication cycle, respectively (see results in **Table 1**), that indicates a fast or slow onset of antiplasmodial activity of the antibiotics.

**Table 1:** Activity of antibiotics against *P. falciparum* strain 3D7 after 3 and 6 days of incubation and *P. knowlesi* strain A1-H.1 after 3 days.

Classe	Drug	Plasmodium falciparum (3D7) and P. knowlesi (A1-H.1) (IC_50_ in nM)				Fold differences between 3 and 6 days
		3 day-assay			6 day-assay	
		3D7		A1-H.1	3D7	
**Tetracyclines**	Chlortetracycline	> 18000			> 18000	NA
	Methacycline	> 18000			3719 ± 3134	NA
	Lymecycline	> 18000			1124 ± 247	NA
	Demeclocycline	> 18000			410.1 ± 165	NA
	Meclocycline	> 18000			291.7 ± 41	NA
	Sarecycline	13378.9 ± 1053			244.1 ± 136	59
	Omadacycline	8443 ± 618			247.8 ± 109	34
**Macrolides**	Oleandomycin	8186 ± 3851			> 18000	NA
	Troleandomycin	> 18000			> 18000	NA
	Josamycin	> 18000			387.8 ± 185	NA
	Boromycin	0.9 ± 0.1	1.3 ± 0.2*	5.9 ± 3.5*	1.0 ± 0.7	0
**Controls**	Minocycline	9393 ± 4354			83.5 ± 25	113
	Eravacycline	2514 ± 550			5.1 ± 1.1	502
	Clindamycin	> 18000			5.9 ± 3.4	NA
	Doxycycline	9579 ± 2756			385.7 ± 292	24
	Chloroquine	8.4 ± 4.9	21.1 ± 11*	14.5 ± 9.3*	10.4 ± 4.8	0

*Measured by SYBR Green-I. All experiments have been done at least 3 times in duplicate.

None of the tetracyclines evaluated in this study showed a pronounced activity (in the nanomolar range) in the 3-day assay against *P. falciparum*, but had some delayed activity after 6 days of drug exposure (**Table 1**). Chlortetracycline, methacycline and lymecycline were still poorly active after 6 days. The first-time tested drugs against *P. falciparum* demeclocycline, sarecycline, omadacycline and the already previously tested drugs/controls doxycycline, minocycline, and eravacycline were active in the nanomolar range against both species after 6 days (**Table 1**). Boromycin was highly active against *P. falciparum* at an IC_50_ of 1.0 nM after 3 days of incubation (**Table 1**), To further characterize its antiplasmodial activity, boromycin was also assessed against *P. knowlesi* and its pronounced activity was confirmed there (**Table 1**).

### 3.2 Cytotoxicity of Boromycin Against Human Cells

Boromycin was cytotoxic at millimolar concentrations against human HepG2 cells with a selectivity index (SI) higher than 200,000. (**Table 2**). Chloroquine was used as comparator and was more cytotoxic than boromycin.

**Table 2:** Cytotoxicity of boromycin and the comparator chloroquine against HepG2 cells and calculated selectivity index.

	IC_50_ (µM) in HepG2 cells	SI 3D7*	SI A1-H.1*
**Boromycin**	1250.5 ± 230	1,388,000	211,864
**Chloroquine**	153.4 ± 52.9	18,214	10,551

*IC_50_ 3-day assays; SI: selectivity index calculated by IC_50_ HepG2/IC_50_ Plasmodium species. All experiments have been done at least 3 times in duplicate.

### 3.3 Characterization of the Antiplasmodial Activity of Boromycin

#### 3.3.1 Activity of Boromycin Against Asexual Stages of *P. falciparum* Chloroquine-Resistant Strains

**Figure 3:**
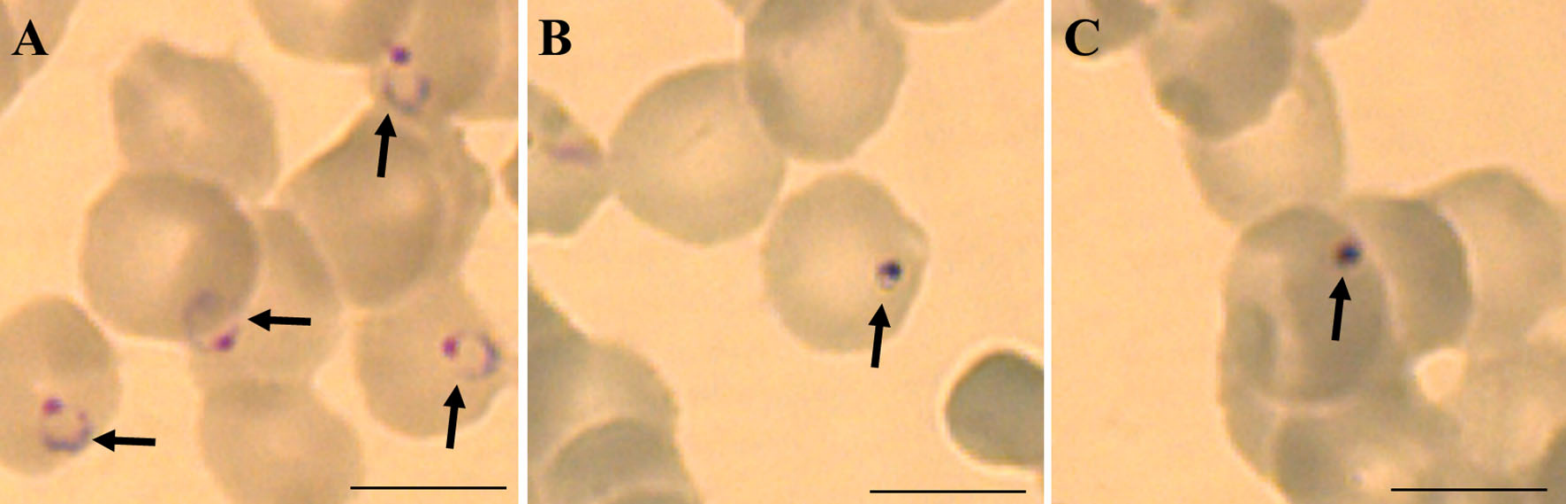
Representative micrographies of *P. falciparum* infected erythrocytes stained with Giemsa: **(A)** untreated ring stage parasites after 6 hours, **(B)** parasites strain 3D7 treated with 1 nM of boromycin for 6 h and **(C)** parasites strain Dd2 treated with 1 nM of boromycin for 6 h. Arrows: parasites. Scale bar: 10 µm.

Different drug resistant strains of *P. falciparum* (Dd2, K1 and 7G8) were incubated with boromycin for 3 days to assess the potential of cross-resistance. Results were comparable to activities against 3D7 with IC_50_ values in the picomolar range, and no cross-resistance to boromycin by these parasite lines was seen (**Table 3**). When investigating the morphological effect of the drug treatment on the parasite, untreated *P. falciparum* parasites of the strain 3D7 (**Figure 3A**) showed the usual ring shape, while boromycin-treated ring-stages (**Figure 3B**), as well as trophozoites and schizonts (– **Supplementary Figure 2**) showed the same pyknotic appearance after boromycin incubation at 1 nM for 6 h. In addition, the same fast effect and morphological changes were also induced in the chloroquine resistant strain Dd2 (**Figure 3C**).

**Table 3:** Antiplasmodial activity of boromycin against different *P.falciparum* chloroquine-resistant strains in comparison to 3D7 after 3 days of incubation.

	Plasmodium falciparum strains (IC_50_ in nM)			
	3D7	Dd2	K1	7G8
**Boromycin**	0.9 ± 0.1	0.6 ± 0.4	0.7 ± 0.1	0.4 ± 0.07
**Chloroquine**	8.4 ± 4.9	302 ± 119	401.4 ± 28.3	303.6 ± 18.2

All experiments have been done at least 3 times in duplicate.

#### 3.3.2 Activity of Boromycin Against Mature Gametocytes

**Figure 4:**
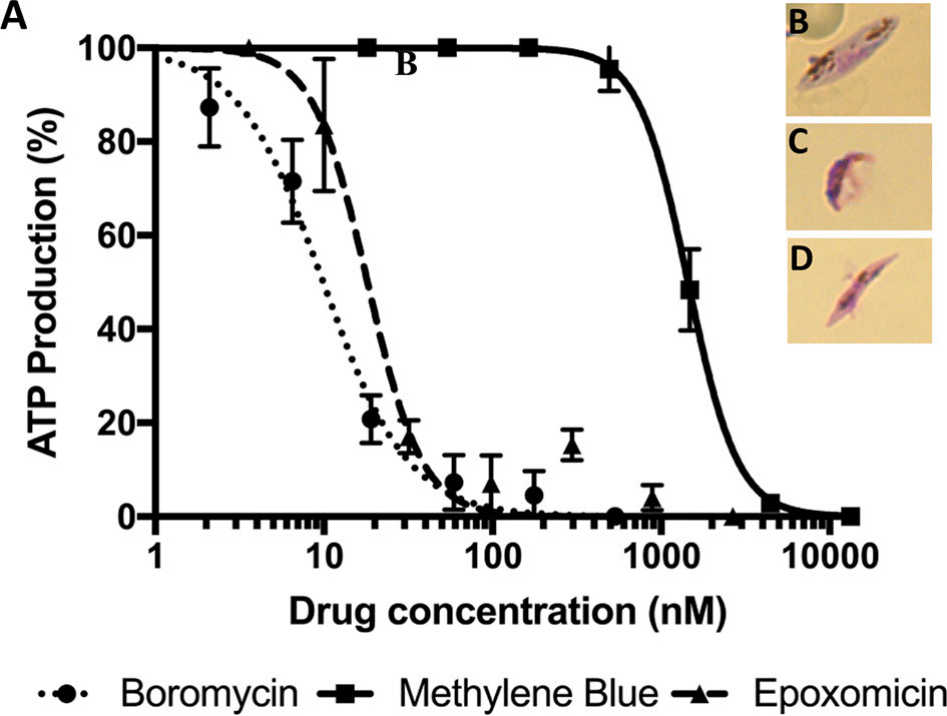
Activity of boromycin, and the positive controls epoxomicin and methylene blue against mature (stage IV-V) *P. falciparum* gametocytes treated for 48 h. **(A)** Boromycin showed a more potent activity than methylene blue and similar activity to epoxomicin. **(B)** Untreated mature gametocytes. **(C)** Boromycin-treated gametocytes and **(D)** epoxomicin-treated parasites. All experiments were done at least 3 times in duplicate.

Mature *P. falciparum* gametocytes (stage IV and V) were incubated with a serial dilution of boromycin for 48 h. Afterwards, ATP production was quantified to evaluate the gametocytocidal effect (**Figure 4A**). The results revealed that boromycin was highly active (IC_50_ at 8.5 ± 3.6 nM), similarly active as the positive comparator epoxomicin (15.2 ± 3.4 nM) and more active than methylene blue (284 ± 35 nM). When observed microscopically, untreated gametocytes displayed their usual elongated shape with rounded edges (**Figure 4A**), but both boromycin (**Figures 4B, C**) and epoxomicin – treated (**Figures 4C, D**) parasites showed thinner body and edges, and intracellular disorganization. The length was not affected in epoxomicin-treated gametocytes, resulting in a needle-like shape, while boromycin-treated gametocytes presented a smaller appearance.

#### 3.3.3 Activity of Boromycin on the Apicoplast

**Figure 5:**
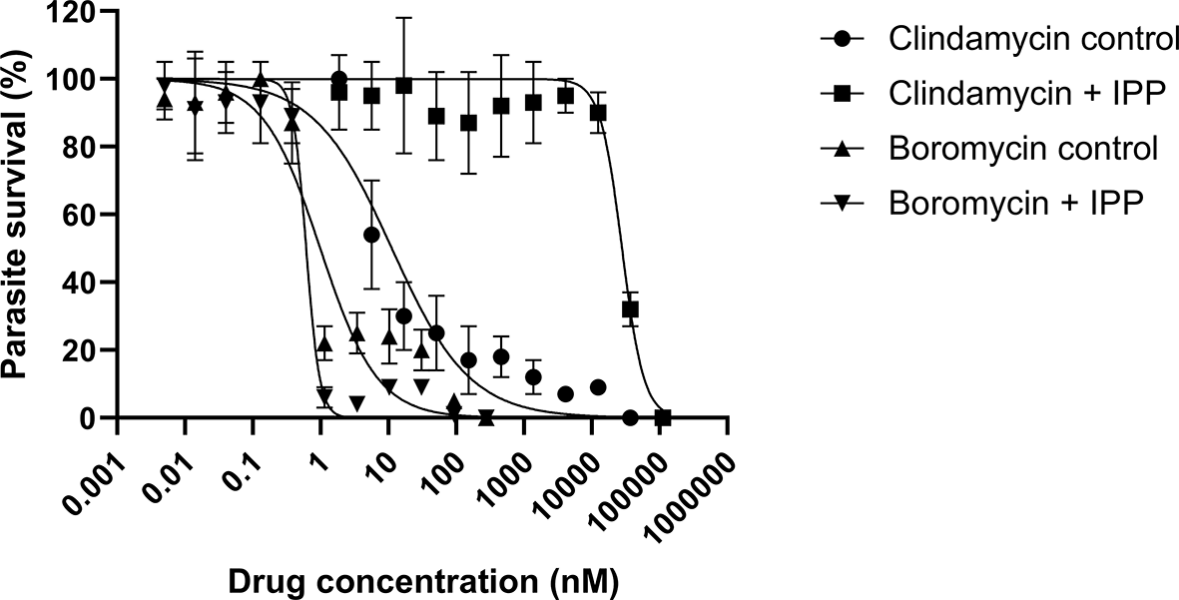
Survival curves of isopentenyl pyrophosphate trilithium salt (IPP) rescue assays. Parasites of the *P. falciparum* strain 3D7 were treated with boromycin or clindamycin (positive control) with or without IPP supplementation and incubated for 6 days. Boromycin-treated parasites were not rescued by IPP, indicating that the apicoplast is not the main target. Experiments were done at least 3 times in duplicate.

*P. falciparum* parasites were incubated with boromycin supplemented with IPP for 6 days to assess whether boromycin targets the apicoplast as described for many antibiotics; clindamycin was used as positive control. At the same time a standard growth inhibition assay without IPP was carried out as a comparator. As expected, clindamycin-treated parasites could be rescued by the supplementation with IPP, but the IC_50_ values for boromycin remained similar at low nanomolar concentrations for both conditions (**Table 4**), suggesting that this drug effect cannot be rescued by IPP and has a different target from the apicoplast (**Figure 5**).

**Table 4:** IC_50_ values obtained with isopentenyl pyrophosphate trilithium salt (IPP) rescue assay in comparison to standard drug assays against *P. falciparum* strain 3D7.

	IC_50_ (nM)		P value (unpaired t-test)
	control	IPP supplementation	
**Clindamycin**	6.9 ± 1.3	>18000	0.02
**Boromycin**	0.9 ± 0.1	1.4 ± 0.8	0.717

All experiments have been done at least 3 times in duplicate.

#### 3.3.4 Ionophoric Activity of Boromycin

*P. falciparum* parasites were incubated with a serial dilution of boromycin, and chloroquine (control) with or without supplementation of 220 µg/mL of MgCl_2_ or KCl, for 3 days to assess whether boromycin has the ionophoric activity on *P. falciparum* as described for *Mycobacterium and Bacillus subtilis* (Abenoja et al., 2021) (**Table 5**). Boromycin and chloroquine displayed the expected IC_50_ value, the supplementation of KCl at 220 µg/mL increased IC_50_ of boromycin by 3-fold, while no difference was observed for chloroquine. On the other hand, the incubation with boromycin or chloroquine in the presence of MgCl_2_ led to a 3-5-fold decrease in the respective IC_50s_.

**Table 5:** Activity of boromycin and the control drug chloroquine against the *P. falciparum* strain 3D7 in the presence/absence of KCl (220 µg/ml) and MgCl_2._ (220 µg/ml).

IC_50_ (in nM)			
	control	KCl supplemented	MgCl_2_ supplemented
**Boromycin**	0.7 ± 0.1	2.1 ± 0.2	0.2 ± 0
**Chloroquine**	12 ± 0.8	11.6 ± 1.4	2.2 ± 0

All experiments have been done at least 3 times in duplicate.

## 4 Discussion

Antibiotics for malaria treatment have been investigated since the middle of the last century, with tetracyclines playing a central role. In recent years, macrolides have also been particularly studied as antimalarials (Gaillard et al., 2015; Gaillard et al., 2016a; Gaillard et al., 2016b). Although the delayed death effect caused by most antibiotics limits their use as first-line chemotherapy, some antibiotics such as doxycycline, clindamycin, and the combination sulfadoxine-pyrimethamine (that does not display the delayed activity) are recommended as prophylaxis or treatment in certain populations (World Health Organization, 2021). The need for new alternative therapies, along with the limited investments in drug discovery to treat malaria, make drug repurposing a worthy alternative. Herein, the *in vitro* antiplasmodial activity of tetracyclines and macrolides not previously tested against Plasmodia along with comparator drugs were evaluated against blood-stages of *P. falciparum* revealing boromycin, as a potent antiplasmodial drug with a fast/first cycle activity that we assessed further.

All tested active tetracyclines showed a delayed death effect against *P. falciparum* (strain 3D7). The newly licensed tetracyclines omadacycline (licensed for treatment of bacterial pneumonia and acute skin infections) (Nuzyra, 2021) and sarecycline (licensed for treatment of acne) (United States Food and Drug Administration, 2021) showed comparable activity to doxycycline. None of the newly tested tetracyclines achieved the activity of the next generation tetracyclines tigecycline (Held et al., 2010) and eravacycline (Koehne et al., 2021) that suggested that modifications at the C9 position on the D ring that circumvents the ribosomal protection resistance mechanism found in bacteria could be beneficial for the activity against Plasmodia. However, the structurally similar omadacycline did not show this improved activity. In a similar way, differences in activities for these three antibiotics were also seen for rickettsia species (Quade et al., 2021). The delayed death effect was also shown by the macrolide josamycin suggesting that the apicoplast could be the target of this drug. The tetracyclines chlortetracycline, methacycline, lymecycline, and the macrolides troleandomycin and oleandomycin were only poorly active with IC_50_ in the micromolar range. Whereas the macrolide boromycin showed a fast onset of activity at a single-digit nanomolar concentration against both *Plasmodium* species.

*P. falciparum* is undoubtably responsible for most of the malaria burden, especially for most of the malaria deaths, which justifies to mainly tailor treatments to this malaria species. However, there are five additional *Plasmodium* species responsible for malaria in humans and when elimination of malaria is the aim, all these species must be targeted. Until recently, *P. falciparum* was the only option for drug screening *in vitro* because the other human malaria parasites could not be kept in continuous *in vitro* culture (Mehlotra et al., 2017; van Schalkwyk et al., 2019). However, now *P. knowlesi* has been adapted for *in vitro* culture and its inclusion in drug screenings could give additional information on antiplasmodial activities of compounds as it is phylogenetically closer to the other human *Plasmodium species* (Rutledge et al., 2017; van Schalkwyk et al., 2019). Even though previous studies have claimed that most antiplasmodial compounds are equipotent against *P. falciparum* and *P. knowlesi* (Moon et al., 2013; Fatih et al., 2013), recent studies have reported different susceptibility profiles (van Schalkwyk et al., 2021) especially for some novel drugs in development. *P. knowlesi* was described as less susceptible to inhibitors of the dihydrofolate reductase, dihydroorotate dehydrogenase (van Schalkwyk et al., 2017), and ATP4 (van Schalkwyk et al., 2019). Herein, we also observed that boromycin was very potent against both species, *P. knowlesi* was slightly less vulnerable than *P. falciparum*, with a 4.5-fold higher IC_50_.

The most promising antibiotic from our screening was the macrolide boromycin. Boromycin was isolated from a *Streptomyces antibioticus* found in a soil sample from Ivory coast and was first described in 1967 (Hütter et al., 1967; Pache and Zaehner, 1969b). It has a potent effect against Gram-positive bacteria *in vitro* (Moreira et al., 2016), and it was shown to be active against HIV (Kohno et al., 1996), *Babesia* (Hütter et al., 1967) and some other pathogens (de Carvalho et al., 2019). Its antiplasmodial activity was already once described in a patent filed in the US in 1973 where its activity in the malaria models of *P. berghei* in mice (ED50: 2.2 mg/kg) and *P. gallinaceum* in birds (ED50: ~ 7 mg/kg) is given (Prelog et al., 1973). Additionally, its *in vitro* activity against *P. falciparum* strain K1 (chloroquine – resistant) and FCR3 (chloroquine – sensitive) was described to be 39 and 40 nM, respectively (Tsutsui et al., 2010). In the same study, only a low selectivity index against MRC-5 cells (~12) was described, but a 97.9% parasite reduction was observed when *P. berghei* – infected mice were treated *p.o*. at 10 mg/kg according to Peter´s test. However, no description of the evaluation of its gametocytocidal activity and stage specific effect could be identified by us.

In our investigation, boromycin showed a high activity and a fast onset of action, a desired property of antimalarials to quickly reduce parasite load and avoid the disease progression to the severe form. It also showed an excellent selectivity index against HepG2 cells. This favorable toxicity profile was also confirmed previously (Moreira et al., 2016), and the LD50 in mice is given as 180 mg/kg^-1^ (Klingel, 1980). These results encouraged a more detailed investigation of the antiplasmodial activity of boromycin.

Boromycin’s *in vitro* activity against laboratory strains showed potent activity as it was active at low nanomolar and even at picomolar concentrations. No cross-resistance with the tested chloroquine/multi-resistant strains (Dd2, 7G8, and K1) was seen as the IC_50_ values were in same range as for the drug sensitive strain 3D7, suggesting that is not affected by the common resistance mechanisms (Ding et al., 2012). Morphologically, ring stage parasites showed a pyknotic appearance after 6 h treatment with boromycin at 1 nM, suggesting that it leads to a rapid death. We additionally evaluated the activity of boromycin against mature gametocytes and could see a very potent activity superior to the positive control methylene blue, similarly active as the proteasome inhibitor epoxomicin. Drugs with transmission-blocking activity are of special interest and would be a great asset for controlling and eliminating malaria (Sinden, 2017; Wadi et al., 2019). Currently, primaquine is the only approved drug active against gametocytes (Lin et al., 2017) but its use is restricted mainly due to its haemolysis-inducing effect in patients with glucose-6-phosphate dehydrogenase deficiency (Recht et al., 2018), and additionally it has to be metabolized by a liver enzyme (CYP2D6) to be active. Drugs with multi-stage activities and novel modes of action are especially desired for the next generation of antimalarials (Ding et al., 2012).

The apicoplast is described as the main target of antibiotics that exert the delayed death effect (Dahl and Rosenthal, 2007; Ekland et al., 2011) and the fast-acting antibiotic fosmidomycin (Uddin et al., 2018). Apicoplast-free parasites can survive and proliferate when the medium is supplemented with IPP (Yeh and DeRisi, 2011), but this did not rescue the boromycin-treated parasites in our assay; therefore, we could rule out the apicoplast as a main target.

The ionophoric activity of boromycin, especially for K+ channels, was firstly described in *Bacillus subtilis* in addition to the impairment of protein, RNA, and DNA synthesis (Pache and Zaehner, 1969a). This ionophoric activity was later confirmed in *Mycobacterium bovis*, where a rapid loss of membrane potential, reduction of intracellular ATP, and leakage of cytoplasmic proteins were induced (Moreira et al., 2016) and the external addition of a high concentration of KCl (20 mg/mL) decreased boromycin activity from 50% to 10%. To assess whether boromycin acts as a potassium ionophore also in *P. falciparum*, parasites were simultaneously incubated with boromycin together with the highest non-toxic concentration (220 µg/mL) of KCl or MgCl_2_ (negative control). We could not incubate *Plasmodium* parasites at a similar high ion concentration as in the *Mycobaterium* experiments, as this concentration was toxic to the parasites. We identified a three-fold IC_50_ increase with the addition of KCl. However, it is most probably not the primary mode of action, as the activity remained in the low nanomolar range. On the other hand, the boromycin IC_50_ decreased with addition of MgCl_2_, this was also seen for chloroquine, an effect that was described before (Hess et al., 1995). Recently, boromycin was shown to decrease intracellular multiplication of both *Toxoplasma gondii* and *Cryptosporidium parvum* (Abenoja et al., 2021), but the potential target in these parasites was not further investigated.

In conclusion, we presented the antiplasmodial activity of different tetracycline and macrolide antibiotics against *P. falciparum*. The tetracyclines tested here did not show higher *in vitro* activities than previously tested tetracyclines and all displayed a slow/second cycle onset of action. The macrolide boromycin showed the most noticeable results as a fast-acting drug against both *Plasmodium* species and *P. falciparum* mature gametocytes. These results, along with the historical antiparasitic effects of boromycin, suggest that boromycin deserves a deeper investigation as an alternative therapy for diseases caused by apicomplexan parasites, including Plasmodia.

## Data Availability Statement

The original contributions presented in the study are included in the article/**Supplementary Material**. Further inquiries can be directed to the corresponding author.

## Author Contributions

JH and LPC designed the study, experiments were performed by SG-O. JH, LPC, and SG-O analyzed the data, all authors contributed to interpretation of the data. The first draft of the manuscript was written by LPC. All authors commented and contributed to the article and approved the final version of the manuscript.

## Conflict of Interest

The authors declare that the research was conducted in the absence of any commercial or financial relationships that could be construed as a potential conflict of interest.

## Publisher’s Note

All claims expressed in this article are solely those of the authors and do not necessarily represent those of their affiliated organizations, or those of the publisher, the editors and the reviewers. Any product that may be evaluated in this article, or claim that may be made by its manufacturer, is not guaranteed or endorsed by the publisher.

## References

[B1] Abenoja J. Cotto-Rosario A. O’Connor R. (2021). Boromycin has Potent Anti-Toxoplasma and Anti-Cryptosporidium Activity. Antimicrob. Agents Chemother. 65, e01278–e01220. doi: 10.1128/AAC.01278-20 33468470 PMC8097477

[B2] Amir A. Cheong F. W. de Silva J. R. Liew J. W. K. Lau Y. L. (2018). Plasmodium Knowlesi Malaria: Current Research Perspectives. Infect. Drug Resist. 11, 1145–1155. doi: 10.2147/IDR.S148664 30127631 PMC6089103

[B3] Balikagala B. Fukuda N. Ikeda M. Katuro O. T. Tachibana S.-I. Yamauchi M.. (2021). Evidence of Artemisinin-Resistance Malaria in Africa. NEJM 385, 1163–1171. doi: 10.1056/NEJMoa2101746 34551228

[B4] Benakis A. Paris M. Loutan L. Plessas C. T. Plessas S. T. (1997). Pharmacokinetics of Artemisinin and Artesunate After Oral Administration in Healthy Volunteers. Am. J. Trop. Med. Hyg. 56, 17–23. doi: 10.4269/ajtmh.1997.56.17 9063354

[B5] Dahl E. L. Rosenthal P. J. (2007). Multiple Antibiotics Exert Delayed Effects Against the Plasmodium Falciparum Apicoplast. Antimicrob. Agents Chemother. 51, 3485–3490. doi: 10.1128/AAC.00527-07 17698630 PMC2043295

[B6] de Carvalho L. P. Kreidenweiss A. Held J. (2021). Drug Repurposing: A Review of Old and New Antibiotics for the Treatment of Malaria: Identifying Antibiotics With a Fast Onset of Antiplasmodial Action. Molecules 26, 2304. doi: 10.3390/molecules26082304 33921170 PMC8071546

[B7] de Carvalho L. P. Sandri T. L. de Melo E. J. T. Fendel R. Kremsner P. G. Mordmüller B.. (2019). Ivermectin Impairs the Development of Sexual and Asexual Stages of Plasmodium Falciparum In Vitro. Antimicrob. Agents Chemother. 63, e00085–e00019. doi: 10.1128/AAC.00085-19 31109978 PMC6658753

[B8] Ding X. C. Ubben D. Wells T. N. C. (2012). A Framework for Assessing the Risk of Resistance for Anti-Malarials in Development. Malar. J. 11, 292–303. doi: 10.1186/1475-2875-11-292 22913649 PMC3478971

[B9] Dunitz J. D. Hawley D. M. Miklol D. White D. N. J. Berlin Y. Marusic R.. (1971). Structure of Boromycin. Helv. Chim. Acta 54, 1709–1713. doi: 10.1002/hlca.19710540624 5131791

[B10] Ekland E. H. Schneider J. Fidock D. A. (2011). Identifying Apicoplast-Targeting Antimalarials Using High-Throughput Compatible Approaches. FASEB J. 25, 3583–3593. doi: 10.1096/fj.11-187401 21746861 PMC3177575

[B11] Fatih F. A. Staines H. M. Siner A. Ahmed M. A. Woon L. C. Pasini E. M.. (2013). Susceptibility of Human Plasmodium Knowlesi Infections to Anti-Malarials. Malar. J. 12, 1–7. doi: 10.1186/1475-2875-12-425 24245918 PMC3874596

[B12] Gaillard T. Dormoi J. Madamet M. Pradines B. (2016a). Macrolides and Associated Antibiotics Based on Similar Mechanism of Action Like Lincosamides in Malaria. Malar. J. 15, 1–11. doi: 10.1186/s12936-016-1114-z 26873741 PMC4752764

[B13] Gaillard T. Madamet M. Pradines B. (2015). Tetracyclines in Malaria. Malar. J. 14, 445. doi: 10.1186/s12936-015-0980-0 26555664 PMC4641395

[B14] Gaillard T. Madamet M. Tsombeng F. F. Dormoi J. Pradines B. (2016b). Antibiotics in Malaria Therapy: Which Antibiotics Except Tetracyclines and Macrolides may be Used Against Malaria? Malar. J. 556, 1–10. doi: 10.1186/s12936-016-1613-y PMC5109779 27846898

[B15] Gardiner D. L. Trenholme K. R. (2015). Plasmodium Falciparum Gametocytes: Playing Hide and Seek. Ann. Transl. Med. 3, 45. doi: 10.3978/j.issn.2305-5839.2015.01.23 25861600 PMC4381475

[B16] Grande E. N. Sanchez A. R. Sanchez F. R. (1956). The Treatment of Malaria With Tetracycline. Antibiot. Med. Clin. Ther. 3, 193–196. 13355230

[B17] Grignard L. Shah S. Chua T. H. William T. Drakeley C. J. Fornace K. M. (2019). Natural Human Infections With Plasmodium Cynomolgi and Other Malaria Species in an Elimination Setting in Sabah, Malaysia. J. Inf. Dis. 220, 1946–1949. doi: 10.1093/infdis/jiz397 31418017 PMC6834065

[B18] Held J. Zanger P. Issifou S. Kremsner P. G. Mordmüller B. (2010). *In Vitro* Activity of Tigecycline in Plasmodium Falciparum Culture-Adapted Strains and Clinical Isolates From Gabon. Int. J. Antimicrob. Agents 35, 587–589. doi: 10.1016/j.ijantimicag.2010.02.003 20227854

[B19] Hess F. I. Kilian A. Söllner W. Nothdurft H. D. Pröll S. Löscher T. (1995). Plasmodium Falciparum and Plasmodium Berghei: Effect of Magnesium on the Development of Parasitemia. Exp. Parasitol. 80, 186–193. doi: 10.1006/expr.1995.1023 7895830

[B20] Hu T. H. Rosli N. Mohamad D. S. A. Kadir K. A. Ching Z. H. Chai Y. H.. (2021). A Comparison of the Clinical, Laboratory and Epidemiological Features of Two Divergent Subpopulations of Plasmodium Knowlesi. Sci. Rep. 11, 20117. doi: 10.1038/s41598-021-99644-8 34635723 PMC8505493

[B21] Hütter R. Keller-Schien W. Knüsel F. Prelog V. Rodgers G. C. Jr. Suter P.. (1967). Stoffwechselprodukte Von Mikroorganismen. 57. Mitteilung. Boromycin. Helv. Chim. Acta 50, 1533–1539. doi: 10.1002/hlca.19670500612 6081908

[B22] Imboden C. A. Cooper W. C. Coatney G. R. Jeffrey G. M. (1950). Studies in Human Malaria. XXIX. Trials of Aureomycin, Chloramphenicol, Penicillin, and Dihydrostreptomycin Against the Chesson Strain of Plasmodium Vivax. J. Natl. Malar. Soc. 9, 377–380. 14804097

[B23] Ishiyama A. Iwatsuki M. Namatame M. Nishihara-Tsukashima A. Sunazuka T. Takahashi Y.. (2011). Borrelidin, a Potent Antimalarial: Stage-Specific Inhibition Profile of Synchronized Cultures of Plasmodium Falciparum. J. Antibiot. 64, 381–384. doi: 10.1038/ja.2011.6 21343942

[B24] Klingel W. (1980). Bor in Biologie, Medizin Und Pharmazie: Physiologische Wirkungen Und Anwendung Von Borverbindungen (Braunschweig: Springer-Verlag), 902.

[B25] Koehne E. Kreidenweiss A. Adegbite B. R. Manego R. Z. McCall M. B. B. Mombo-Ngoma G.. (2021). *In Vitro* Activity of Eravacycline, a Novel Synthetic Halogenated Tetracycline, Against the Malaria Parasite Plasmodium Falciparum. J. Glob. Antimicrob. Resist. 24, 93–97. doi: 10.1016/j.jgar.2020.11.024 33301999

[B26] Kohno J. Kawahata T. Otake T. Morimoto M. Mori H. Ueba N.. (1996). Boromycin, an Anti-HIV Antibiotic. Biosci. Biotechnol. Biochem. 60, 1036–1037. doi: 10.1271/bbb.60.1036 8695905

[B27] Lelièvre J. Almela M. J. Lozano S. Miguel C. Franco V. Leroy D.. (2012). Activity of Clinically Relevant Antimalarial Drugs on Plasmodium Falciparum Mature Gametocytes in an ATP Bioluminescence “Transmission Blocking” Assay. PloS One 7, 1–8. doi: 10.1371/journal.pone.0035019 PMC3325938 22514702

[B28] Lin J. T. Lon C. Spring M. D. Sok S. Chann S. Ittiverakul M.. (2017). Single Dose Primaquine to Reduce Gametocyte Carriage and Plasmodium Falciparum Transmission in Cambodia: An Open-Label Randomized Trial. PloS One 12, e0168702. doi: 10.1371/journal.pone.0168702 28591198 PMC5462369

[B29] Mehlotra R. K. Blankenship D. Howes R. E. Rakotomanga T. A. Ramiranirina B. Ramboarina S.. (2017). Long-Term In Vitro Culture of Plasmodium Vivax Isolates From Madagascar Maintained in Saimiri Boliviensis Blood. Malar. J. 16, 1–13. doi: 10.1186/s12936-017-2090-7 29100506 PMC5670718

[B30] Mendes A. M. Albuquerque I. S. Machado M. Pissarra J. Meireles P. Prudêncio M. (2017). Inhibition of Plasmodium Liver Infection by Ivermectin. Antimicrob. Agents Chemother. 61, 1–8. doi: 10.1128/AAC.02005-16 PMC5278742 27895022

[B31] Milner D. A. Jr. Whitten R. O. Kamiza S. Carr R. Liomba G. Dzamalala C.. (2014). The Systemic Pathology of Cerebral Malaria in African Children. Front. Cell Infect. Microbiol. 4, 1–13. doi: 10.3389/fcimb.2014.00104 25191643 PMC4139913

[B32] Mishra S. J. Liu W. Beebe K. Banerjee M. Kent C. N. Munthali V.. (2021). The Development of Hsp90β-Selective Inhibitors to Overcome Detriments Associated With Pan-Hsp90 Inhibition. J. Med. Chem. 11, 1545–1557. doi: 10.1021/acs.jmedchem.0c01700 PMC8996186 33428418

[B33] Moon R. W. Hall J. Rangkuti F. Ho Y. S. Almond N. Mitchell G. H.. (2013). Adaptation of the Genetically Tractable Malaria Pathogen Plasmodium Knowlesi to Continuous Culture in Human Erythrocytes. Proc. Natl. Acad. Sci. U. S. A. 110, 531–536. doi: 10.1073/pnas.1216457110 23267069 PMC3545754

[B34] Moreira W. Aziz D. B. Dick T. (2016). Boromycin Kills Mycobacterial Persisters Without Detectable Resistance. Front. Microbiol. 7, 199. doi: 10.3389/fmicb.2016.00199 26941723 PMC4761863

[B35] Noedl H. Bronnert J. Yingyuen K. Attlmayr B. Kollaritsch H. Fukuda M. (2005). Simple Histidine-Rich Protein 2 Double-Site Sandwich Enzyme-Linked Immunosorbent Assay for Use in Malaria Drug Sensitivity Testing. Antimicrob. Agents Chemother. 49, 5–8. doi: 10.1128/AAC.49.8.3575-3577.2005 PMC1196223 16048989

[B36] Nsanzabana C. (2019). Resistance to Artemisinin Combination Therapies (ACTs): Do Not Forget the Partner Drug! Trop. Med. Infect. Dis. 4, 26. doi: 10.3390/tropicalmed4010026 PMC6473515 30717149

[B37] Nuzyra. Available at: https://www.nuzyra.com/hcp/ (Accessed October 15, 2021).

[B38] Otoguro K. Ui H. Ishiyama A. Kobayashi M. Togashi H. Takahashi Y.. (2003). *In Vitro* and *In Vivo* Antimalarial Activities of a Non-Glycosidic 18-Membered Macrolide Antibiotic, Borrelidin, Against Drug-Resistant Strains of Plasmodia. J. Antibiot. 56, 727–729. doi: 10.7164/antibiotics.56.727 14563165

[B39] Pache W. Zaehner H. (1969a). Metabolic Products of Microorganisms. Arch. für. Mikrobiol. 67, 156–165. doi: 10.1007/BF00409681 4247775

[B40] Pache W. Zaehner H. (1969b). Stoffwechselprodukte Von Mikroorganismen. Arch. Mikrobiol. 288, 281–288. doi: 10.1007/BF00412060 4988748

[B41] Pluijm R. W. Imwong M. Chau N. H. Hoa N. T. Thuy-Nhien N. T. Thanh N. V.. (2019). Determinants of Dihydroartemisinin-Piperaquine Treatment Failure in Plasmodium Falciparum Malaria in Cambodia, Thailand, and Vietnam: A Prospective Clinical, Pharmacological, and Genetic Study. Lancet Inf. Dis. 19, 952–961. doi: 10.1016/S1473-3099(19)30391-3 PMC6715822 31345710

[B42] Portugaliza H. P. Miyazaki S. Geurten F. J. A. Pell C. Rosanas-Urgell A. Janse C. J.. (2020). Artemisinin Exposure at the Ring or Trophozoite Stage Impacts Plasmodium Falciparum Sexual Conversion Differently. Elife 9, e60058. doi: 10.7554/eLife.60058 33084568 PMC7577739

[B43] Prelog V. Zaehner H. Bickel H. (1973). U.S. Patent No 584,215 (Ardsley, New York: Ciba-Geigy Corporation).

[B44] Quade B. R. Ramírez-Hernández A. Blanton L. S. (2021). *In Vitro* Susceptibility of Rickettsia Species to Eravacycline, Omadacycline, and Tigecycline. Antimicrob. Agents Chemother. 65, e0066521. doi: 10.1128/AAC.00665-21 34060898 PMC8284447

[B45] R Core Team. (2014). R: A Language and Environment for Statistical Computing (Vienna, Austria: R Foundation for Statistical Computing). Available at: http://www.R-project.org.

[B46] Recht J. Ashley E. A. White N. J. (2018). Use of Primaquine and Glucose-6-Phosphate Dehydrogenase Deficiency Testing: Divergent Policies and Practices in Malaria Endemic Countries. PloS Negl. Trop. Dis. 12, e0006230. doi: 10.1371/journal.pntd.0006230 PMC5908060 29672516

[B47] Ribaut C. Berry A. Chevalley S. Reybier K. Morlais I. Parzy D.. (2008). Concentration and Purification by Magnetic Separation of the Erythrocytic Stages of All Human Plasmodium Species. Malar. J. 7, 1–5. doi: 10.1186/1475-2875-7-45 18321384 PMC2292734

[B48] Ritz C. Streibig J. C. (2005). Bioassay Analysis Using R. J. Stat. Softw. 12, 1–22. doi: 10.18637/jss.v012.i05

[B49] Rutledge G. G. Böhme U. Sanders M. Reid A. J. Cotton J. A. Maiga-Ascofare O.. (2017). Plasmodium Malariae and P. Ovale Genomes Provide Insights Into Malaria Parasite Evolution. Nature 542, 101–104. doi: 10.1038/nature21038 28117441 PMC5326575

[B50] Siciliano G. Alano P. (2015). Enlightening the Malaria Parasite Life Cycle: Bioluminescent Plasmodium in Fundamental and Applied Research. Front. Microbiol. 6, 1–8. doi: 10.3389/fmicb.2015.00391 26029172 PMC4426725

[B51] Sinden R. E. (2017). Developing Transmission-Blocking Strategies for Malaria Control. PloS Pathog. 13, 1–12. doi: 10.1371/journal.ppat.1006336 PMC5500365 28683121

[B52] Straimer J. Gnädig N. F. Witkowski B. Amaratunga C. Duru V. Ramadani A. P.. (2015). K13-Propeller Mutations Confer Artemisinin Resistance in Plasmodium Falciparum Clinical Isolates. Science 347, 428–431. doi: 10.1126/science.1260867 25502314 PMC4349400

[B53] Tsutsui A. Furuya Y. Hirose T. Kim R. Masuma R. Matsumoto A.. (2010). Boromycin Derivatives: Synthesis and Antimalarial Activity *In Vitro* and In Vivo. Heterocycles 82, 289–295. doi: 10.3987/COM-10-S(E)55

[B54] Uddin T. Mcfadden G. I. Goodman C. D. (2018). Validation of Putative Apicoplast-Targeting Drugs Using a Chemical Supplementation Assay in Cultured Human Malaria. Antimicrob. Agents Chemother. 62, e01161–e01117. doi: 10.1128/AAC.01161-17 PMC5740311 29109165

[B55] United States Food and Drug Administration Drug Trials Snapshot: Seysara. Available at: https://www.fda.gov/drugs/drug-approvals-and-datab (Accessed October 15, 2021).

[B56] van Biljon R. Niemand J. van Wyk R. Clark K. Verlinden B. Abrie C.. (2018). Inducing Controlled Cell Cycle Arrest and Re-Entry During Asexual Proliferation of Plasmodium Falciparum Malaria Parasites. Sci. Rep. 8, 1–14. doi: 10.1038/s41598-018-34964-w 30409996 PMC6224408

[B57] van Schalkwyk D. A. Blasco B. Davina N. R. Liew J. W. K. Amir A. Lau Y. L.. (2019). Plasmodium Knowlesi Exhibits Distinct *In Vitro* Drug Susceptibility Profiles From Those of Plasmodium Falciparum. Int. J. Parasitol. Drugs Drug Resist. 9, 93–99. doi: 10.1016/j.ijpddr.2019.02.004 30831468 PMC6403410

[B58] van Schalkwyk D. A. Moon R. W. Blasco B. Sutherland C. J. (2017). Comparison of the Susceptibility of Plasmodium Knowlesi and Plasmodium Falciparum to Antimalarial Agents. J. Antimicrob. Chemother. 72, 3051–3058. doi: 10.1093/jac/dkx279 28961865 PMC5890772

[B59] van Schalkwyk D. A. Moon R. W. Duffey M. Leroy D. Sutherland C. J. (2021). *Ex Vivo* Susceptibility to New Antimalarial Agents Differs Among Human-Infecting Plasmodium Species. Int. J. Parasitol. Drugs Drug Resist. 17, 5–11. doi: 10.1016/j.ijpddr.2021.07.002 34315108 PMC8327131

[B60] Wadi I. Nath M. Anvikar A. R. Singh P. Sinha A. (2019). Recent Advances in Transmission-Blocking Drugs for Malaria Elimination. Future Med. Chem. 11, 3047–3088. doi: 10.4155/fmc-2019-0225 31782936

[B61] White N. J. (2008). Plasmodium Knowlesi: The Fifth Human Malaria Parasite. Clin. Infect. Dis. 15, 172–173. doi: 10.1086/524889 18171246

[B62] World Health Organization. Guidelines for Malaria. Available at: https://www.who.int/publications/i/item/guidelines-for-malaria (Accessed Acessed December 10, 2021).

[B63] World Health Organization. (2021). World Malaria Report. Available at: https://www.who.int/teams/global-malaria-programme/reports/world-malaria-report-2021 (Accessed December 22, 2021).

[B64] WWARN K13 Genotype-Phenotype Study Group. (2019). Association of Mutation in the Plasmodium Falciparum Kelch13 Gene (Pf3D7_1343700) With Parasite Clearance Rates After Artemisinin-Based Treatments – A WWARN Individual Patient Data Meta-Analysis. BMC Med. 17, 1. doi: 10.1186/s12916-018-1207-3 30651111 PMC6335805

[B65] Yeh E. DeRisi J. L. (2011). Chemical Rescue of Malaria Parasites Lacking an Apicoplast Defines Organelle Function in Blood-Stage Plasmodium Falciparum. PloS Biol. 9, e1001138. doi: 10.1371/journal.pbio.1001138 21912516 PMC3166167

