# Supplementary material for: Boromycin has Rapid-Onset Antibiotic Activity Against Asexual and Sexual Blood Stages of Plasmodium falciparum
Source: Front Cell Infect Microbiol. 2022 Jan 14;11:802294. doi: 10.3389/fcimb.2021.802294 (PMC8795978; doi:10.3389/fcimb.2021.802294)
Supplement: Supplementary file 1 [file DataSheet_1.docx]

Supplementary information

Supplementary Figure 1: Survival curves of *P. falciparum* strain 3D7 supplemented with MgCl_2_ or KCl for 72 h. The experiments were done at least three times in duplicate.

Supplementary Figure 2: Representative micrographies of *P. falciparum* infected erythrocytes stained with Giemsa: (A) untreated trophozoites, (B) boromycin-treated trohpozoites at 1 nM for 6 h, (C) untreated schizonts and (D) boromycin-treated schizonts for 6 h. Arrows: parasites. Scale bar: 10 µM. At least three biological replicates were observed.

Supplementary Table 1: List of drugs used in this study.

| **Antibiotic**  **class** | **Name of antibiotic** | **Solvent** | **Company** | **MW in**  **g/mol** |
| --- | --- | --- | --- | --- |
| Tetracyclines | Metacycline | DMSO | SCBT | 442.42 |
|  | Demeclocycline | water | SCBT | 464.85 |
|  | Lymecycline | water | SCBT | 602.63 |
|  | Meclocycline | DMSO | CC | 476.9 |
|  | Sarecycline hydrochloride | DMSO | Adooq Bioscience | 524 |
|  | Omadacycline | DMSO | Hycultec | 593,11 |
| Macrolides | Oleandomycin | DMSO | SCBT | 687.86 |
|  | Boromycin | DMSO | SCBT | 879.87 |
|  | Josamycin | Ethanol | SCBT | 827.99 |
|  | Troleandomycin | DMSO | SCBT | 813.97 |
| Controls | Chloroquine diphosphate | distilled water | SA | 515.86 |
|  | Clindamycin | DMSO | SA | 479.46 |
|  | Doxycycline Hyclate | water | SA | 512.94 |
|  | Minocycline  hydrochloride | water | SA | 457.48 |
|  | Eravacycline | DMSO | Hycultec | 558.56 |
|  | Epoxomicin | DMSO | SA | 554.72 |
|  | Methylene Blue | distilled water | SA | 319.85 |

SA: Sigma Aldrich, CC: Cayman Chemicals, SCBT: Santa Cruz Biotechnology, DMSO: dimethylsulfoxide.
