# Supplementary figures and images for: Boromycin has Rapid-Onset Antibiotic Activity Against Asexual and Sexual Blood Stages of Plasmodium falciparum
Source: Front Cell Infect Microbiol. 2022 Jan 14;11:802294. doi: 10.3389/fcimb.2021.802294 (PMC8795978; doi:10.3389/fcimb.2021.802294)

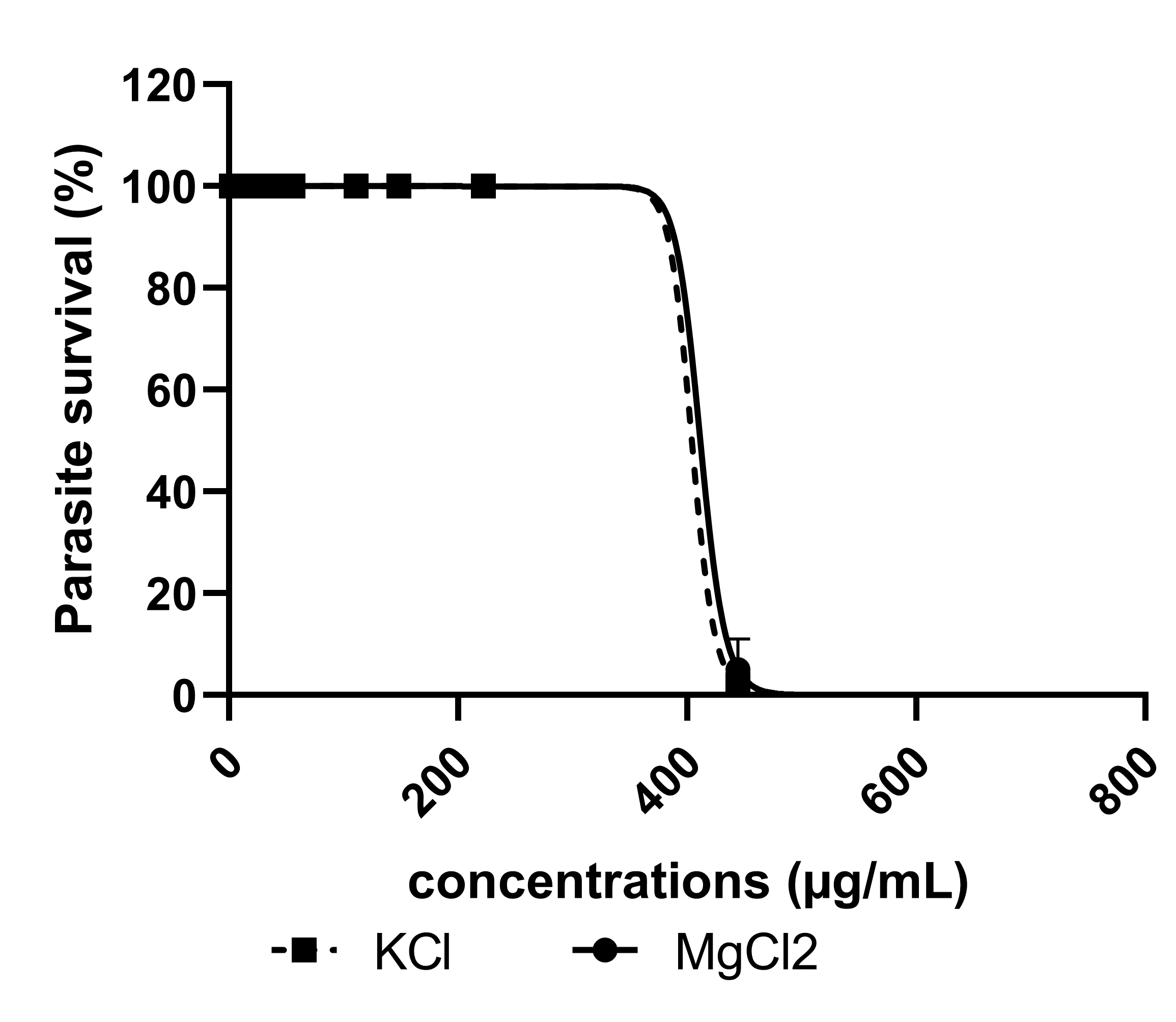

Supplement: Supplementary file 2 [file Image_1.tif]

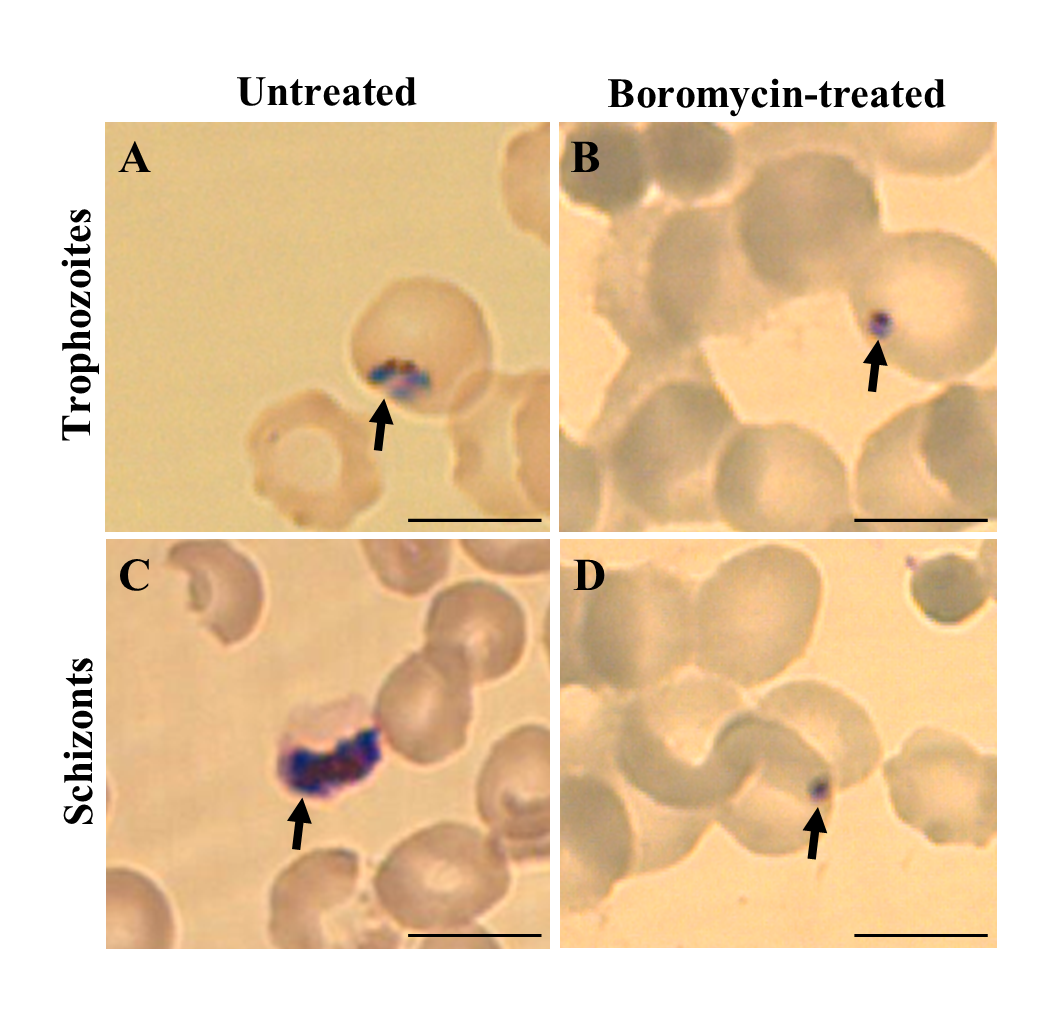

Supplement: Supplementary file 3 [file Image_2.tif]
